# Successful sirolimus therapy of an aplastic anemia patient with chronic kidney disease: A case report

**DOI:** 10.1097/MD.0000000000020669

**Published:** 2020-06-05

**Authors:** Haiyue Niu, Weiwei Qi, Yihao Wang, Limin Xing, Rong Fu, Zonghong Shao, Huaquan Wang

**Affiliations:** Department of Hematology, General Hospital, Tianjin Medical University, Tianjin, China.

**Keywords:** aplastic anemia, chronic kidney disease, drug therapy, sirolimus

## Abstract

**Rationale:**

It is very difficult to treat patients with aplastic anemia accompanied by chronic kidney disease. The nephrotoxicity of cyclosporine limits its use in these patients. Most of these patients also lack suitable sibling donors. Sirolimus, as a new type of immunosuppressive agent, has good therapeutic effect, lower toxicity, especially lower nephrotoxicity, thus attracting the attention of hematologists.

**Patient concerns:**

This 55-year-old Chinese male patient suffered from pancytopenia and renal insufficiency and has a poor quality of life.

**Diagnosis:**

The patient was diagnosed as severe aplastic anemia with chronic kidney disease-G3a.

**Interventions:**

We started the sirolimus therapy with the initial dose of 1 mg per day. Based on the good tolerability and clinical effect, we increased the dose of sirolimus to 2 mg per day after 2 weeks.

**Outcomes:**

By taking sirolimus, the patient's peripheral blood cell count gradually increased, and he achieved blood transfusion independent, and eventually the blood cell count was completely normal.

**Lessons:**

We consider that sirolimus is a safe, effective, and well-tolerated oral drug that can be used as a treatment for aplastic anemia patients with chronic kidney disease.

## 1 Introduction

Aplastic anemia (AA) is a bone marrow (BM) failure syndrome defined as pancytopenia with hypocellular marrow, and without abnormal infiltration and reticulin proliferation. The acquired AA is immune mediated disorder with the destruction of hematopoietic stem cells and progenitor cells by active T lymphocytes.^[1]^ Allogeneic hematopoietic stem cell transplantation (allo-HSCT) from an HLA identical sibling donor and immune-suppressive therapy (IST) are the first-line treatments for AA. IST with antithymocyte globulin (ATG)/antilymphocyte globulin (ALG) and cyclosporine A (CsA) is recommended for AA patients older than 40 years or without a suitable donor.^[2]^ It is difficult to treat AA patient with chronic kidney disease (CKD). In this report, we describe the clinical course of an SAA patient with renal failure who was successfully treated by sirolimus.

Sirolimus (Rapamycin), a macrolide antibiotic, inhibits the serine–threonine kinase mTOR, and blocks CsA-resistant and calcium-independent pathways late in the progression of the T-cell cycle in contrast to the calcineurin inhibitors, CsA and FK506, which act earlier and only on calcium-dependent pathways.^[3]^

## 2 Case report

**Figure 1:**
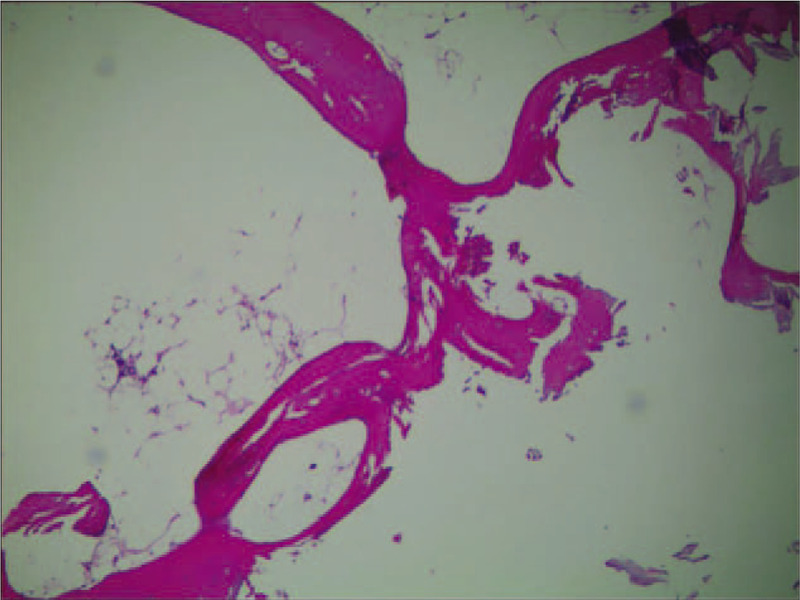
Bone marrow biopsy at the time of the first diagnosis showed severe hypoplasia.

A 55-year-old male presented in March 2017 with fatigue and pancytopenia. Laboratory studies revealed a white blood cell count (WBC) of 0.8×10^9^/L, neutrophil cell count of 0.48 × 10^9^/L, platelet (Plt) count of 53 × 10^9^/L, hemoglobin (Hb) concentration of 58 g/L, and reticulocytes at only 3.3 × 10^9^/L (The normal reference value of medical indexes above are 3.5–9.5 × 10^9^/L, 1.8–6.3 × 10^9^/L, 125–350 × 10^9^/L, 130–175 g/L, and 24–84 × 10^9^/L, respectively). The serum anti-nuclear antibody and rheumatoid factor were negative. BM biopsy revealed severe hypoplasia (Fig. 1). The BM smear demonstrated 20% cellularity (myeloid, 24.5%; erythroid, 64.5%; lymphocytes, 8%, and some plasma cells and tissue basophils). The cytogenetics of the BM mononuclear cells revealed 46 XY. BM mononuclear cell antibody, Plt antibody and paroxysmal nocturnal hemoglobinuria clone by flow cytometry were all negative. The percentages of CD59 negative leukocytes and red cells in peripheral blood were in the normal range. The immune phenotype and FISH about myelodysplastic syndrome were negative. The subsequent myelodysplastic syndrome -related genes next generation sequence was completed for the patient, and no positive gene mutation was found. According to the Camitta criteria,^[4]^ the diagnosis of AA must be reached at least 2 of the followings: Hb<100 g/L, Plt <50 × 10^9^/L, neutrophil count<1.5 × 10^9^/L. We further assessed the severity following the modified Camitta criteria,^[4]^ and the patient was up to the standard of SAA (marrow cellularity<25% (or 25%–50% with<30% residual hematopoietic cells), plus at least 2 of the followings: 1.reticulocytes count <20 × 10^9^/L, Plt <20 × 10^9^/L, neutrophil count<0.5 × 10^9^/L). He had a history of CKD secondary to chronic glomerulonephritis for 20 years. The levels of serum creatinine, BUN and uric acid were 115 umol/L, 10.9 mmol/L, and 461 umol/L, respectively (the upper limit of the normal values is 115, 8.3, and 414umol/L, respectively).Estimated glomerular filtration rate was 46 mL/min/1.73 m^2^ meeting the 2012 modified kidney disease: improving global outcomes (KDIGO) standard^[5]^ of CKD-G3a (estimated glomerular filtration rate ranges from 45 to 59 mL/min/1.73 m^2^).Finally, He was diagnosed with SAA with CKD-G3a.

Initially, the patient was given 20 mg/d prednisone. The peripheral blood counts recovered rapidly with stable count of Hb fluctuating between 110 and 130 g/L, and the percentage of reticulocytes rising from 0.61% to more than 6%. The prednisone was gradually tapered to 7.5 mg/d 4 months later. The patient was admitted to our hospital again because of diarrhea with bicytopenia (WBC count of 6.22 × 10 ^9^/L, Plt count of 90 × 10 ^9^/L, Hb concentration of 89 g/L).Even worse, the renal function further deteriorated with the level of creatinine rising from 352 to 649 umol/L, the level of BUN rising from 30 to 50 mmol/L and the uric acid maintaining a relatively high level of 505 to 656 umol/L, which alarmed that dialysis was necessary. Considering the severe symptoms of edema and respiratory distress, we arranged the patient to take hemodialysis once every other day to maintain the levels of creatinine at approximately 250 to 400 umol/L. The patient presented with progressive multiple lineage reduction as well, and required frequent transfusions of Plt and RBC to maintain Plt level at 20 × 10^9^/L and Hb concentration at 80 g/L. The total amount transfused RBC and Plt were 10 and 86 units, respectively. Although we tried to stimulate hematogenesis by increasing the dose of prednisone and using some drugs such as recombined human erythropoietin, recombined human thrombopoietin to stimulus hematopoietical cells, peripheral blood cell counts failed to recover but even worsened so that he was still RBC and Plt transfusion dependent.

Considering the nephrotoxicity of CsA, we prescribed sirolimus with an initial dose of 1 mg per day. Dramatically, the peripheral blood counts showed an upward trend and the patient achieved transfusion independence a week later. Another week passed, given that the patient could tolerate the main toxicities such as oral mucositis and infection well, we increased the dose of sirolimus to 2 mg per day. WBC and Plt were recovered completely (WBC of 6.7 × 10^9^/L; Hb of 85 g/L; Plt of 116 × 10^9^/L) in the third week after sirolimus therapy.

**Figure 2:**
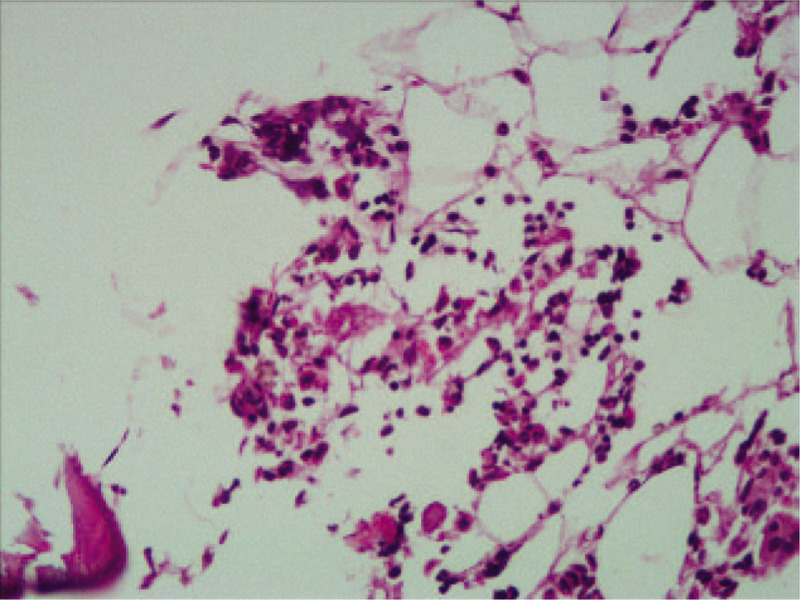
Bone marrow biopsy after sirolimus therapy revealed granulocyte, erythrocyte, and megakaryocyte were all hyperplasia.

One year later, the patient was in stable situation with Hb of 132 g/L; RBC of 4.15 × 10^12^/L; the absolute number of neutrophils of 5.68 × 10^9^/L; and Plt of 185 × 10^9^/L. The BM smear showed that granulocyte, erythrocyte and megakaryocyte were all hyperplasia (myeloid, 58.5%; erythroid, 25.5%; lymphocytes, 12%), so did BM biopsies (Fig. 2). The patient continued to use sirolimus 2 mg per day and accepted regular dialysis twice a week. Up to now, the total follow-up was 35 months and this patient is still being followed up closely. Except the renal function (The count of serum creatinine, BUN and uric acid were 793 umol/L, 20.9 mmol/L, and 442 umol/L, respectively), the hematological disease has been alleviated.

The patient has provided informed consent for publication of the case.

## 3 Discussion

How to treat AA patient with CKD remains a difficult medical problem. On one hand, drug therapy may aggravate the renal function; on the other hand, abnormal renal function may alter the pharmacokinetics and/or pharmacodynamics of drug, which puts forward more stringent requirements on the dosage of the drug. Hiraga et al reported a SAA patient, of whom needed hemodialysis and transfusion, being treated successfully with ATG.^[6]^ They also highlighted the importance of iron chelation therapy for such kind of people requiring repeated transfusion. Hamaki et al reported a clinical case of a SAA patient with renal failure requiring hemodialysis, was treated successfully by taking allo-HSCT from his HLA-identical sibling. The conditional regimen of this case included melphalan, horse ATG and total lymphoid irradiation that had low nephrotoxicity.^[7]^ Still, successful treatment experience and pharmacokinetic data of AA with renal insufficiency requiring regular hemodialysis is limited.

Most AA patients are treated with CsA and ATG/ALG that is the most classical IST currently. When CsA was long-term applied, the toxic and side effects of CsA cannot be ignored. Even worse, there is limited successful treatment experience on allo-HSCT in AA with renal failure which could provide reference for clinician when they make diagnosis and treatment decisions. Based on these dilemmas, there are many studies aimed at finding effective alternative or adjuvant treatments.

He et al had successful experience of using sirolimus combined with CsA when treating 2 cases of relapsed/refractory SAA patients, which provided us a paradigm.^[8]^ For this SAA patient we discussed above with a 20-year history of CKD, he received sirolimus alone as an alternative treatment for CsA. And this was the first case reported about successful sirolimus therapy of an AA patient with CKD.

Sirolimus is a novel macrolide immunosuppressant with better curative effect, lower toxicity, especially lower nephrotoxicity. From the perspective of clinical application, sirolimus has a better anti-rejection effect and a good synergistic effect with immunosuppressive agents such as CsA and tacrolimus (FK506). One study had compared CsA with sirolimus in improving peripheral blood counts, ameliorating composition of BM cell and prolonging the survival rate of immune-mediated AA mouse models. It showed that both CsA and sirolimus alleviated the efficacy of mouse AA model immune-mediated BM failure.^[9]^ But they own different mechanism. CsA increases the cytoplasmic nuclear factor of activated T cell-1 after T cell receptor was stimulated, while sirolimus inhibits 2 key signaling molecules in the mTOR pathway, S6 kinase and protein Phosphorylation of kinase B. The main mechanisms of sirolimus are as follows:^[10]^ first, it can reduce inflammatory cytokines of Th1 such as interferon-γ and tumor necrosis factor-α effectively. Second, it can increase the cytokine of Th2, such as interleukin-10 and stimulate functional regulatory T cell proliferating. A recent study showed that, the expression of Foxp3 in regulatory T (Treg) cells of SAA mouse models was decreased, and the apoptosis of Treg cells were significantly increased, which was speculated that the low expression of Foxp3 causes autoimmune imbalance.Foxp3 expression was up-regulated and apoptosis was decreased in spleen Treg cells in the sirolimus group.^[11]^ Although we have made some progress in sirolimus therapy of AA, the current evidence does not support that sirolimus synergizing with cyclosporine could improving therapeutic effect. A randomized controlled trial of 77 AA patients, 35 of whom were added sirolimus on a standard ALG combined with CsA basis treatment, did not show the superiority of sirolimus.^[12]^ The preventive effect of sirolimus on long-term use of CsA in patients with severe recurrence risk of AA is currently under investigation.

**Figure 3:**
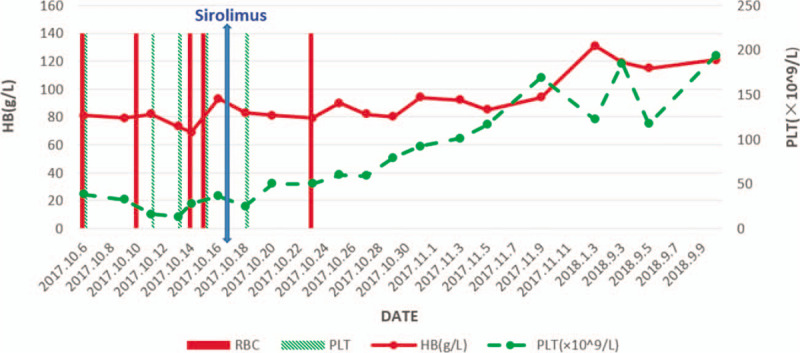
The variation trend of hemoglobin and plateletand the frequency of blood transfusions.

mTOR plays an important role in cell senescence, tumor, immunity, brain function, muscle development, lipid production, and homeostasis, and glucose homeostasis.^[13]^ This case of SAA patient with CKD-G3a requiring hemodialysis has complete recovery of peripheral blood counts and has achieved transfusion independence (Fig. 3).

In summary, we consider that sirolimus is a kind of safe and well tolerated oral drug that may be used as an alternative or adjuvant therapy for AA patients with CKD requiring hemodialysis. Although adding sirolimus on a standard ALG combined with CsA treatment did not show better efficacy, the efficacy of single drug with sirolimus is still unknown, especially for the AA patients with renal insufficiency. The time of adding sirolimus remains to be determined. There is no doubt that these questions still require a large number of well-designed randomized controlled trials or clinical cases to provide evidence-based medicine.

## Author contributions

**Conceptualization:** Huaquan Wang.

**Data curation:** Haiyue Niu, Weiwei Qi, Yihao Wang, Limin Xing, Rong Fu.

**Funding acquisition:** Huaquan Wang, Zonghong Shao.

**Writing – original draft:** Haiyue Niu.

**Writing – review & editing:** Huaquan Wang.
